# Transcriptomic Exploration of Tetrahydrocurcumin Effects in Chronic Kidney Disease

**DOI:** 10.3390/biomedicines14071457

**Published:** 2026-06-26

**Authors:** Alyssa Mariana Alvarez, Winston Hibler, Su Mi Lee, Mahyar Khazaeli, Han Liu, Tiffany Tran, Jie Wu, Yitong Zhao, Catherine Huynh, Bhupinder Singh, Wei Ling Lau

**Affiliations:** 1 Department of Biomedical Engineering, University of California, Irvine, CA 92697, USA; 2 Department of Medicine, Division of Nephrology, University of California, Irvine, CA 92697, USA; winstonhibler22@gmail.com (W.H.); sumilee@dau.ac.kr (S.M.L.); han_liu2000@hotmail.com (H.L.); tiffany.tran7@westernu.edu (T.T.); yitonz5@hs.uci.edu (Y.Z.); cvhuynh22@gmail.com (C.H.); bsinghmdllc@gmail.com (B.S.); 3 Department of Internal Medicine, College of Medicine, Dong-A University, Busan 49201, Republic of Korea; 4 Department of Pathology, Kaiser Permanente Baldwin Park Medical Center, Baldwin Park, CA 91706, USA; mahyar.khazaeli@gmail.com; 5 Department of Biological Chemistry, University of California, Irvine, CA 92697, USA; jiew5@uci.edu

**Keywords:** chronic kidney disease, rat nephrectomy model, tetrahydrocurcumin, transcriptomics

## Abstract

**Introduction:** Chronic kidney disease (CKD) involves a progressive loss of renal function and is characterized by chronic oxidative stress and kidney fibrosis. Tetrahydrocurcumin (THCu), a metabolite of curcumin, may possess antioxidant benefits in CKD. This study evaluated the transcriptomic changes and therapeutic potential of THCu against kidney damage and fibrosis in the 5/6 nephrectomy rat CKD model. **Methods:** Adult female Sprague–Dawley rats were randomized into CKD groups and three THCu doses were tested (100, 300 and 500 mg/kg). A liposomal formulation of THCu was given twice daily via oral gavage for 4 weeks. Serum creatinine and proteinuria were measured, and kidney fibrosis was assessed on histology. Kidney lysates were processed for total RNA sequencing to analyze differential gene expression in the experimental groups. The data were screened for outliers prior to ANOVA and correlation analyses. **Results:** In the untreated CKD group, serum creatinine and proteinuria were increased compared to control animals. Transcriptomic profiling revealed that untreated CKD animals exhibited marked upregulation across three key gene categories: immune cell activation, kidney injury and fibrosis, and inflammation and oxidative stress. THCu treatment mitigated these pathways by which there was downregulation of markers of immune cell activation as well as the kidney injury marker *Kim1*, while the fibrosis markers *Col1a1* and *Col3a1* were decreased to expression levels similar to non-CKD control animals. Furthermore, the highest dose of THCu at 500 mg/kg triggered a cellular detoxification and metabolic clearance response, with highly significant upregulation of *Abcb11* and *Gls2*. Antioxidant benefit was evidenced by upregulation of *Gpx1* in the high-dose THCu group compared to the untreated CKD group. Pathway enrichment analysis demonstrated that the high-dose THCu group restored key metabolic and signaling pathways disrupted in renal fibrosis, including small and organic solute metabolism, fatty acid oxidation, lipid biosynthesis, and peptide hormone response. Furthermore, the treatment upregulated essential anion and organic solute transport functions. Proteinuria was reduced with THCu therapy; however, serum creatinine and urine creatinine clearance were not significantly modified in comparison to untreated CKD rats. **Conclusions:** Oral THCu therapy demonstrated promising transcriptional changes in antioxidant and anti-fibrotic pathways in a rat CKD model. Confirmatory protein-level studies are needed to clarify benefits on kidney function.

## 1. Introduction

Chronic kidney disease (CKD) is a heterogeneous group of diseases that are characterized by the progressive loss of renal function over time. Kidney fibrosis is a hallmark of CKD pathogenesis and progression, whereby renal function progressively declines with the accumulation of scar tissue [1,2,3,4]. Several pathophysiological mechanisms influence the development of kidney fibrosis, including chronic low-grade inflammation, oxidative stress, hypertension, and apoptosis [5,6,7,8]. Consequently, therapeutic interventions for CKD often target pro-fibrotic pathways [9,10,11,12].

Curcumin (1,7-bis [4-hydroxy-3-methoxyphenyl]-1,6-heptadiene-3,5-dione; diferuloylmethane) is the chief bioactive curcuminoid in turmeric (*Curcuma longa*), a plant product widely used in culinary applications. Medicinal benefits that have been reported with curcumin include antioxidant [13,14,15], anti-inflammatory [16], antidiabetic [17,18], and antifibrotic [19] effects. Paradoxically, curcumin was also reported to exhibit a minor dose-dependent degree of pro-oxidant activity, which dampened enthusiasm for its use as a therapeutic agent [20,21].

Tetrahydrocurcumin (THCu) is the principal metabolite of curcumin and exists in a fully reduced state, which prevents THCu from oxidizing other molecules in redox reactions, thereby conferring a better safety profile than curcumin. Further, THCu may surpass curcumin in terms of antioxidant potency [22] and anti-inflammatory effects [23,24].

A key limitation to the practical application of curcuminoids is their poor oral bioavailability. Curcuminoids are inefficiently absorbed through the small intestine, then rapidly metabolized and excreted upon reaching the bloodstream [25]. Several strategies have been developed to improve the oral bioavailability of curcuminoids, such as co-administration with the glucuronidase inhibitor piperine [26] or complexing curcumin with a carrier molecule such as lecithin [27]. A novel carrier approach is to encapsulate curcuminoids within nanoparticles for transcellular delivery using liposomal formulations [28,29] or poly(lactic-co-glycolic acid) nanoparticles [30], both of which improve curcuminoid bioavailability to similar extents as piperine and lecithin.

Our lab previously reported that THCu incorporated into a pelleted diet ameliorated renal fibrosis and hypertension in 5/6-nephrectomized CKD rats [11] in conjunction with modulation of kidney fibrosis pathways. THCu amplified antioxidant scavenging enzymes, specifically superoxide dismutase (SOD), catalase, and glutathione peroxidase (GPX), independent of nuclear Nrf2 translocation. In the current exploratory study, we evaluated oral delivery of a proprietary liposomal THCu formulation to investigate transcriptomic changes in the same partial nephrectomy rat CKD model. An oral route was chosen for its translational relevance to future clinical trials.

## 2. Materials and Methods

### 2.1. Rat CKD Model

**Figure 1:**
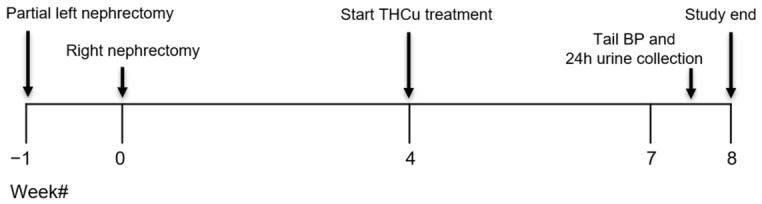
Experimental timeline. BP: blood pressure; THCu: tetrahydrocurcumin.

All experimental protocols were approved by the University of California, Irvine Institutional Committee for the Use and Care of Experimental Animals (protocol #AUP-21-157). Adult female Sprague–Dawley rats (body weight 225–250 g) were randomized to control or 5/6-nephrectomy CKD. Females were chosen over males due to their tendency to develop greater proteinuria and renal fibrosis in a 5/6-nephrectomy model than males [31]. The housing conditions were standard and as follows: temperature range from 68 to 79 °F, 12 h light–dark cycle, food and water ad libitum, standard solid-bottom cages with group-housing. Experimental groups were as follows: control (CTL, *n* = 6), untreated CKD (*n* = 10), CKD + THCu 100 mg/kg (T100, *n* = 10), CKD + THCu 300 mg/kg (T300, *n* = 10), and CKD + THCu 500 mg/kg (T500, *n* = 10). CKD was induced via 2-stage surgeries under isoflurane anesthesia: the two poles of the left kidney were resected, followed by total nephrectomy of the right kidney one week later. Mortality rate was 5/40 (12.5%) and was attributed to anesthesia or bleeding issues within 72 h after surgery (*n* = 3 after surgery #1, *n* = 1 after surgery #2) and severe uremia (*n* = 1 in T100 group). Therefore, the number of animals included in final analyses were CTL (*n* = 6), CKD (*n* = 9), T100 (*n* = 8), T300 (*n* = 9), and T500 (*n* = 9). Buprenorphine analgesia, 0.05 mg/kg, was given via intraperitoneal injection at the start of each surgery. THCu treatment began four weeks after the right nephrectomy and was given for a total of four weeks. Tail blood pressure measurements (Coda System, Kent Scientific, Torrington, CT, USA) and 24 h urine collections in individual metabolic cages (Tecniplast, West Chester, PA, USA) were done within one week prior to study termination. The experimental timeline is shown in Figure 1.

### 2.2. Tetrahydrocurcumin (THCu) Treatment

To improve bioavailability, THCu was administered as a liposomal formulation composed of 10% *w*/*w* THCu, 5% Captex 300, 20% Phosal 75 SA and 65% Labrasol ALF (Hub Therapeutics LLC, Phoenix, AZ, USA). Four weeks after the second surgery (right nephrectomy), CKD rats were randomized to untreated CKD vs. THCu treatment groups. Liposomal THCu therapy was administered as twice-daily oral gavage for four weeks, and three doses were tested. The stock liposomal THCu concentration was 105.12 mg/mL and was diluted in drinking water immediately before dosing for total volume up to 2.5 mL. Serum samples via tail vein blood draw were taken two hours after the oral gavage dose in the first week of THCu therapy to analyze blood THCu levels.

### 2.3. Tissue Harvest and Biochemical Assays

After four weeks on the assigned therapies, rats were euthanized by exsanguination via cardiac puncture under inhaled isoflurane anesthesia. Serum was aliquoted and kidney tissues were collected for histology and RNA analysis. Blood urea nitrogen (BUN) and creatinine (Cr) were analyzed with colorimetric kits from BioAssay Systems (Hayward, CA, USA), with 30× dilution used for urine creatinine. Total urine protein from 24 h urine collection was measured using the Rat Urinary Protein Assay Kit (Chondrex Inc., Redmond, WA, USA). Creatinine clearance was calculated as follows:

$${\mathrm{CrCL}}_{\mathrm{wt}}=\frac{U_{\mathrm{Cr}}\;\times \;U_{V}}{S_{\mathrm{Cr}}\;\times \;1440\;\times \;W}$$

with CrCL_wt_ being the weight-normalized creatinine clearance (mL/min × kg), U_Cr_ is the urine creatinine concentration (mg/dL or µmol/L), U_V_ is the total urine volume collected over 24 h (mL), S_Cr_ is the serum creatinine concentration (mg/dL or µmol/L), 1440 is the total time of the collection period (minutes in 24 h), and W is the rat’s body weight (kg).

### 2.4. Mass Spectrometry to Measure Serum THCu Levels

Serum THCu levels were measured at the Mass Spectrometry facility at University of California, Irvine’s Chemistry Department with modifications based on our published protocol [11]. A 50 µL aliquot of serum was treated with 150 µL of 1000 U of beta-glucuronidase/sulfatase (EC 3.2.1.31) from Helix pomatia (catalog# G7017-1ML, Sigma-Aldrich, St. Louis, MO, USA) in water. The mixture was vortexed and incubated at 37 °C for one hour to hydrolyze the phase-2 conjugates of curcuminoids. After incubation, curcuminoids were extracted with 1 mL of ethyl acetate containing d6-THCu 10 ng/mL internal standard (THCu with 6 deuterated hydrogens, MW 378), and the mixture was vortexed for one minute. The mixture was subjected to centrifugation at 15,000× *g* for six minutes at 4 °C and the upper organic layer was transferred to a 2 mL microcentrifuge tube and evaporated to dryness at 45 °C in a Speed Vac. The dried extract was reconstituted in 200 µL of 25% acetonitrile. Tetrahydrocurcumin (MW 372) reference standard fluka 50202-10MG was purchased from Sigma-Aldrich. A stock 20 mg/mL solution was prepared with acetonitrile, and dilutions were prepared with deionized water to generate a standard curve ranging 1–1000 ng/mL. Standards and prepared samples were injected (10 µL) into the HPLC-MS/MS instrument, Waters Quattro Premier XE equipped with UPLC (Waters^TM^, Milford, MA, USA). The UPLC has a BEH C18 column, which allows rapid sample throughput. Mobile phase A was 2% acetonitrile with 0.2% acetic acid, and mobile phase B was 100% acetonitrile with 0.2% acetic acid. Analysis was performed using multiple reaction monitoring MS/MS with standard calibration. The THCu transition was m/z 373.0 → 136.8.

### 2.5. Kidney Histology

For histologic assessment of fibrosis, kidney tissues were fixed in 10% neutral buffered formalin and routinely processed for paraffin embedding. Paraffin sections of kidney (5 µm thickness) were deparaffinized with xylene, dehydrated in alcohol series, and stained with Masson’s trichrome, then examined under a photomicroscope (Nikon Eclipse, Tokyo, Japan). Six to eight animals were studied from each group, with three images captured at 10× objective per animal. An ImageJ (v1.54g) macro was used for quantification of kidney fibrosis (% area stained blue on Masson’s trichrome) by an investigator blinded to the study groups [11]. Average fibrosis scores were compared across groups.

### 2.6. Kidney mRNA Sequencing

Kidney total mRNA from five animals per experimental group were submitted to the UC Irvine Genomics Research and Technology Hub (GRTH) for sequencing on the Illumina HiSeq4000 platform. Quality control on raw FASTQ files was performed using FastQC (v0.11.7) [32]. Low-quality reads and adapter sequences were excluded using Trimmomatic (v0.35) [33]. The cleaned reads were then mapped to the Ensembl rnor_6.0 reference genome using the HISAT2 aligner (v2.1.0) [34]. Transcripts were assembled with StringTie (v1.3.4d) [35] with transcript assembly restricted to only outputting the transcripts that matched the reference transcripts. The prepDE.py function of StringTie was subsequently used to generate the gene counts matrix for downstream analysis. All software tools involved in pre-processing the RNA reads were accessed through the High-Performance Computing (HPC) Cluster at the University of California, Irvine. All analytical tools following pre-processing were accessed through Bioconductor or GitHub (v3.8.4) and utilized in RStudio (v1.3.1073).

### 2.7. Bulk RNA Deconvolution and Design of Analytical Framework

Since whole tissue lysate was used for RNA extraction, cell-type heterogeneity was assessed using a single-cell mouse kidney dataset from Wu and colleagues (there is no available single-cell reference dataset specific to rat kidney) [36]. Orthologous genes between mice and rats were obtained from the Rat Genome Database [37] then used to reannotate genes with murine gene symbols in order to match with the single-cell reference. Cell-type proportions comprising each sample were then estimated using BisqueRNA (v1.0.5) [38]. Preliminary quality control multidimensional scaling (MDS) plots showed that the lane replicates for sample D6 (from T300 group) were substantially different from one another (leading logFC separation >1 in dimension 1 and >5 in dimension 2 which could have been due to library preparation variations or instrument artifacts, Figure S1), thus the sample was excluded from the analysis.

### 2.8. Differential Expression Analysis

RStudio (v1.3.1073) was used with R (v4.0.2) to access packages via Bioconductor [39]. Cell-type deconvolution showed strong cell-type heterogeneity across samples, as well as within sample replicates. We applied limma-voom (v3.16.2) [40,41] within the edgeR [42] package to account for replicate-level variation through the voomLmFit() function, which used correlation statistics between lane replicates as a blocking factor together with observation-level precision weights while accounting for loss of degrees of freedom [43].

### 2.9. Gene Ontology, Functional Enrichment, and Pathway Analysis

ClusterProfiler [44] was accessed via Bioconductor in RStudio. ClusterProfiler was used to conduct Gene Ontology overexpression analysis for both models. Significant overexpression was defined to be an adjusted *p*-value ≤ 0.05 after applying the Benjamini–Hochberg multiple testing correction. The NIH Database for Annotation, Visualization, and Integrated Discovery (DAVID) was used to perform KEGG pathway analysis. For Disease Ontology analysis, genes were queried in the Rat Genome Database (RGD) to find gene lists that were the most relevant to our parameters of interest.

### 2.10. Statistical Analysis

Data was screened for outliers using the Grubbs’ test, and Bartlett’s test was used to assess homogeneity of variances across groups. For datasets with equal variances, group data were analyzed using one-way ANOVA with post hoc Tukey, and *p* < 0.05 was considered significant. For nonparametric data, Kruskal–Wallis analysis was used (*p* < 0.05 considered significant) with Dunn’s Multiple Comparison Test. Data are reported as mean ± SEM. Figures were generated using GraphPad Prism software (v11.0.2, GraphPad Software, San Diego, CA, USA).

## 3. Results

### 3.1. Serum and Urine Data

Table 1 summarizes serum and urine chemistries and BP data in CTL and CKD animals. CKD animals showed a significant increase in serum creatinine and BUN, and decreased urine creatinine clearance compared with CTL rats. They also exhibited significant proteinuria: average 24 h urine protein 95.9 mg/day compared with 1.2 mg/day in the CTL group. THCu was not detected in the serum from CTL and untreated CKD animals. Serum THCu level (measured two hours after oral gavage dose) increased proportionally with increasing THCu doses of T100, T300, and T500. Proteinuria was attenuated with THCu treatment especially with the T100 dose; differences between treatment groups were not statistically significant. Markers of kidney function (BUN, creatinine, and urine creatinine clearance) were not significantly altered with THCu therapy.

### 3.2. Kidney Histology Results

**Figure 2:**
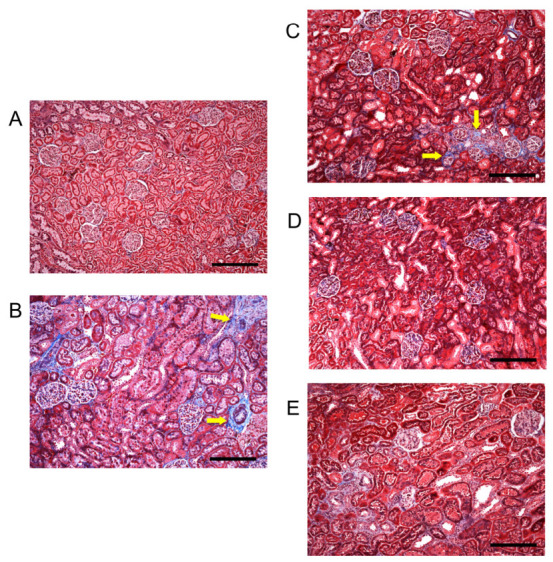
Representative images of kidney fibrosis (stained blue, yellow arrows) on Masson’s Trichrome stain; 10× objective, scale bar = 200 μm. (**A**) = CTL, (**B**) = CKD, (**C**) = CKD + T100, (**D**) = CKD + T300, (**E**) = CKD + T500 where T100 = tetrahydrocurcumin, 100 mg/kg, twice per day; T300 = tetrahydrocurcumin, 300 mg/kg, twice per day; T500 = tetrahydrocurcumin, 500 mg/kg, twice per day.

Masson’s trichrome was used to visually assess percent area of kidney fibrosis (stained blue). Figure 2 shows representative kidney histology from the experimental groups. Fibrosis ranged 0.06–2.5% of the total analyzed area and was increased 3.7-fold in untreated CKD compared with CTL rats. There was a trend for decreased fibrosis area with THCu therapy; however, this did not reach statistical significance.

### 3.3. Kidney Total mRNA Sequencing Results

**Figure 3:**
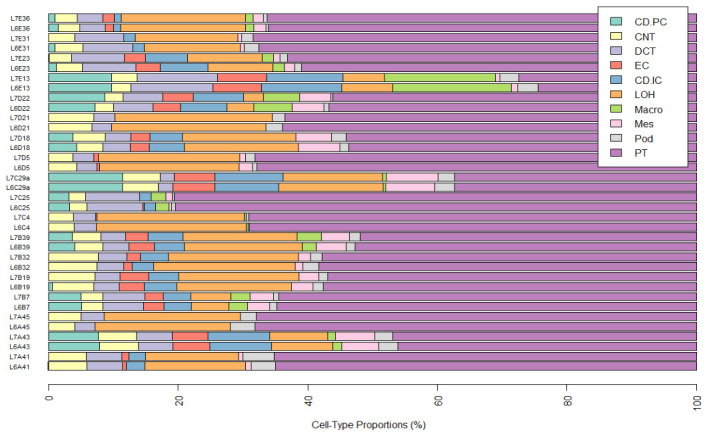
Bar plot demonstrating cell types that were detected in kidney total RNA samples using BisqueRNA. CD.PC: principal cells of collecting duct; CNT: connecting tubule; DCT: distal collecting tubule; EC: endothelial cells; CD.IC: intercalated cells of collecting duct; LOH: loop of Henle; Macro: macrophages; Mes: mesangial cells; Pod: podocytes; PT: proximal tubule.

Bulk RNA deconvolution using BisqueRNA characterized cell-type composition across samples, as well as a more subtle variance within samples (Figure 3). Adopting the methods employed by Kong et al. [45], principal component regression was conducted on cell-type proportions and then correlated to the principal components (PCs) of the expression matrix (Figure S2). Significant cell-type PCs were defined to have correlation −log_10_(*p*) > 2 (*p* < 0.01) and explain at least 0.1% of total variance, rounded to the nearest 0.01%. The majority cell types captured were proximal tubule and loop of Henle cells.

**Figure 4:**
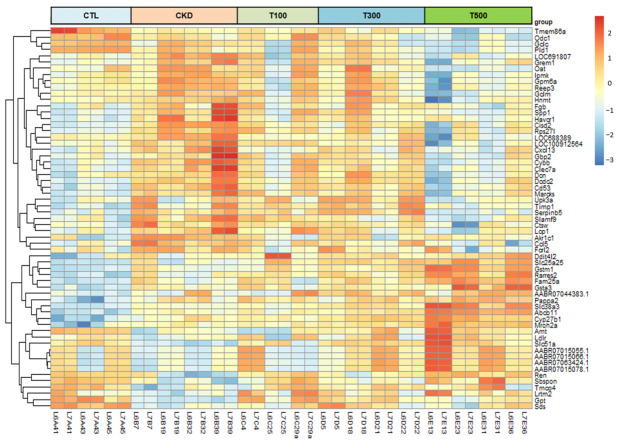
Heatmap of differentially expressed genes (DEGs) across control (CTL), chronic kidney disease (CKD) and tetrahydrocurcumin treatment groups. T100 = tetrahydrocurcumin, 100 mg/kg, twice per day; T300 = tetrahydrocurcumin, 300 mg/kg, twice per day; T500 = tetrahydrocurcumin, 500 mg/kg, twice per day.

Differentially expressed genes (DEGs) were defined to have an adjusted *p* < 0.05 and fold-change > 1.5 (|log_2_FC| > 0.6). Linear regression modeling using the limma-voom pipeline showed a substantial increase in the number of DEGs when comparing CKD vs. CTL. With adjustment for heterogeneity in cell-type composition between samples, there was a dose-dependent increase in the number of DEGs in THCu treatment groups compared to untreated CKD (Table 2). Figure 4 demonstrates the heatmap of DEGs across experimental groups.

**Figure 5:**
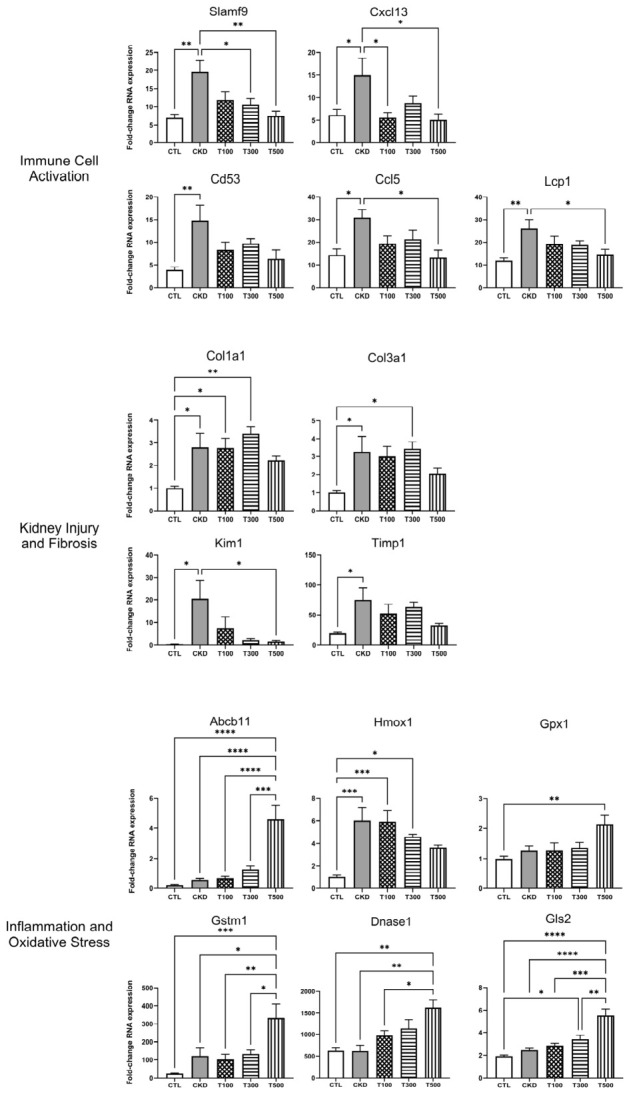
Kidney mRNA fold-change expression of genes related to immune cell activation, kidney injury and fibrosis, and inflammation and oxidative stress in the 5/6-nephrectomized rat model. Experimental groups include control (CTL), untreated chronic kidney disease (CKD), and CKD treated with liposomal tetrahydrocurcumin at 100, 300, and 500 mg/kg (T100, T300, T500). Gene abbreviations: Immune cell activation: Slamf9 (SLAM family member 9), Cxcl13 (C-X-C motif chemokine ligand 13), Cd53 (CD53 molecule), Ccl5 (C-C motif chemokine ligand 5), and Lcp1 (lymphocyte cytosolic protein 1). Kidney injury and fibrosis: Col1a1 (collagen type I alpha 1 chain), Col3a1 (collagen type III alpha 1 chain), Kim1 (kidney injury molecule-1), and Timp1 (tissue inhibitor of metalloproteinases 1). Inflammation and oxidative stress: Abcb11 (ATP binding cassette subfamily B member 11), Hmox1 (heme oxygenase 1), Gpx1 (glutathione peroxidase 1), Gstm1 (glutathione S-transferase mu 1), Dnase1 (deoxyribonuclease 1), and Gls2 (glutaminase 2). * *p* < 0.05, ** *p* < 0.01, *** *p* < 0.001, and **** *p* < 0.0001.

To investigate CKD pathways of interest, we examined transcriptomic changes in genes relevant to kidney injury and oxidative stress (Figure 5). Compared to the CTL group, the untreated CKD rats exhibited a marked upregulation of genes associated with immune cell activation as well as kidney injury and fibrosis. Notably, immune markers such as *Slamf9, Lcp1, Cd53, Ccl5,* and *Cxcl13* were highly elevated in the untreated CKD group. THCu treatment downregulated these genes compared to untreated CKD. Furthermore, *hepatitis A virus cellular receptor 1* (*Havcr1*), also known as *Kidney injury molecule-1* (*Kim1*), a well-established biomarker of renal tubular damage [46,47,48], was significantly increased in CKD. *Kim1* expression was downregulated across the THCu-treated groups with only the T500 group demonstrating significant reduction. Kidney fibrosis genes, including *Col1a1* and *Col3a1*, and the tissue remodeling marker *Timp1*, were significantly upregulated in CKD, and were reduced to levels similar to CTL following T500 therapy.

Among the inflammatory and oxidative stress markers, *Heme Oxygenase-1 (Hmox1)* was significantly upregulated in the untreated CKD group relative to healthy controls. THCu treatment reduced *Hmox1* expression, suggesting mitigation of oxidative stress. Consistent with prior reports on the antioxidant properties of curcuminoids, the T500 dose induced an upregulation of the antioxidant enzyme *glutathione peroxidase (Gpx1)* by approximately 70% compared to the untreated CKD group. Furthermore, T500 uniquely triggered a significant upregulation of xenobiotic clearance and metabolic stress response genes, including *Abcb11, Gstm1, Dnase1,* and *Gls2*. Because these genes were not significantly different between CTL and untreated CKD animals, their robust induction suggests a pharmacological effect of high-dose THCu.

**Figure 6:**
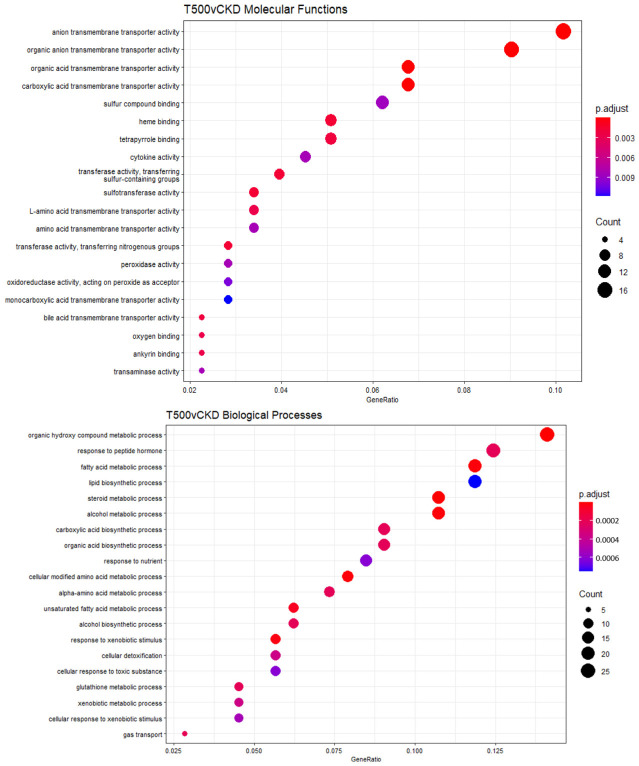
Functional enrichment dot plots of Gene Ontology terms for biological processes (**top**) and molecular functions (**bottom**) for the high dose tetrahydrocurcumin group (T500) compared with untreated CKD animals.

Unbiased functional enrichment analysis revealed that broader networks governing metabolic and oxidative stress were profoundly disrupted in CKD, and the high dose T500 group demonstrated the most significant transcriptomic changes among the three THCu groups. Therefore, we focused downstream analyses on T500 vs. CKD. Functional enrichment analysis confirmed the strong influence of THCu on metabolic pathways related to oxidative stress (Figure 6). Of note, small and organic solute metabolism were downregulated in CKD and was restored with T500 treatment. T500 therapy was also associated with improved fatty acid metabolism and lipid biosynthesis, compared to untreated CKD animals.

## 4. Discussion

Our findings provide a molecular foundation for the protective effects of THCu on CKD progression, consistent with our prior report [11]. THCu treatment attenuated CKD-induced proteinuria, with the most pronounced reduction observed in the T100 group (however, proteinuria levels across the three THCu groups were not statistically different). Transcriptomic profiling revealed that the untreated CKD group exhibited marked upregulation in genes related to immune cell activation, kidney injury and fibrosis, and inflammation and oxidative stress. THCu treatment mitigated these pathways by suppressing markers driving immune cell activation and renal fibrosis with a notable reduction in *Slamf9, Ccl5, Cxcl13* and *Kim1*, while the fibrosis markers *Col1a1* and *Col3a1* were decreased to expression levels similar to CTL. The T500 group triggered an antioxidant and cellular detoxification response with highly significant upregulation of *Abcb11*, *Gls2* and *Gpx1*. Pathway enrichment analysis further demonstrated that the T500 group restored key metabolic and signaling pathways that are usually disrupted and downregulated in CKD progression.

The pronounced transcriptomic changes observed in gene expression in the T500 group revealed key signaling pathways underlying THCu therapy. As demonstrated in Figure 5, CKD was associated with upregulation of genes important in immune cell activation (*Slamf9, Lcp1, Cd53, Ccl5,* and *Cxcl13*) and kidney fibrosis and injury (*Col1a1, Col3a1, Kim1* and *Timp1*). THCu treatment was noted to suppress several immune cell activation markers as well as *Kim1*, which is a key marker for kidney injury. Its significant downregulation in the THCu-treated groups, particularly with the highest dose (T500), suggests that THCu mitigated tubular injury. It is important to note that the effect of THCu on fibrosis genes (*Col1a1, Col3a1*) was modest at the 4-week time point, which suggests that reversing existing fibrotic transcription may require prolonged targeted treatment. Consistent with prior reports on the antioxidant properties of curcuminoids [11,49], THCu strongly influenced genes related to oxidative stress. THCu treatment led to the upregulation of the antioxidant enzyme *Gpx1* by approximately 70% in the T500 group compared to the untreated CKD group. Conversely, the cytoprotective stress-response gene *Hmox1* was substantially induced in the untreated CKD group, and was ameliorated with THCu treatment (likely reflecting lowered oxidative stress although protein-level studies will be necessary to confirm the delicate balance of oxidant/antioxidant mediators). T500 treatment induced highly significant upregulation of xenobiotic clearance and metabolic stress response genes (*Abcb11, Gstm1, Dnase1,* and *Gls2*). *Abcb11* is a gene commonly associated with the liver; however, it is also expressed in the kidney proximal tubule and may function as a urinary efflux pump [50]. *Abcb11* and *Gstm1* govern cellular efflux [51] and detoxification [52], while *Gls2* regulates metabolic energy shifts [53] and *Dnase1* clears extracellular debris [54]. This unique transcriptomic signature indicates that while high dose THCu maximizes immune suppression and antioxidant defense, it also triggers cellular detoxification and metabolic clearance.

This metabolic shift is strongly supported by the functional enrichment analysis (Figure 6), which revealed that T500 treatment restored critical metabolic and signaling pathways typically lost during renal fibrosis. Small and organic solute metabolism was the most significantly impacted biological process category, driven largely by the organic hydroxy compound metabolic process. In CKD, these pathways are severely downregulated as tubular cells lose their functional identity and undergo dedifferentiation, leading to renal fibrosis and functional decline [55]. Upregulation of these pathways with T500 treatment suggests restoration of the kidney’s clearance capacity.

The enrichment analysis also highlighted a significant upregulation in the response to peptide hormone pathway. As CKD progresses, dedifferentiation of renal tubular cells leads to epithelial-to-mesenchymal transition and dysregulation of response to systemic endocrine signals [56,57]. The prominent enrichment of this pathway indicates beneficial THCu effects in preserving tubular epithelial cell response to physiological hormones, required for maintaining overall homeostasis. The T500 treatment also corrected disruptions in cellular energy by improving the fatty acid metabolic process and the lipid biosynthesis process. In CKD, impaired fatty acid oxidation and accumulation of toxic lipids (collectively known as lipotoxicity) contributes to chronic inflammation and fibrosis [58,59]. By upregulating these pathways, T500 restores the kidney’s ability to properly synthesize necessary lipids and utilize fats for energy, thereby mitigating lipid-mediated tissue damage.

Gene Ontology enrichment analysis of molecular functions indicated that anion and organic solute transport was the most significantly impacted category (Figure 6). In CKD, anion and organic solute transport is severely downregulated as tubular cells are damaged and lose their specialized transport proteins. This transport failure promotes uremic toxin accumulation and systemic toxicity [60]. Further, disruptions in carboxylic acid transport are linked to mitochondrial dysfunction and tubular cell death [61]. T500 treatment induced expression of genes relevant to transport of anions, organic solutes and carboxylic acid, concurrent with decreased proteinuria. The improved tubular transport and mitochondrial function will need to be confirmed in future studies.

Our findings are consistent with previous studies showing that THCu administration is associated with attenuated fibrosis in cardiac [62], renal [11,49], and hepatic [63] in vivo disease models. Both curcumin and THCu exhibit broad pharmacological activities against fibrosis pathways, with THCu emerging as a promising alternative due to superior anti-inflammatory effects [23,24] and lower risk of paradoxical pro-oxidant activity. This current study used liposomal THCu administered via oral gavage twice daily for a total of four weeks. This administration achieved dose-proportional serum levels of 112 ± 27 ng/mL (T100), 1010 ± 292 ng/mL (T300), and 5505 ± 3631 ng/mL (T500) at two hours post-dose. In comparison, our 2018 study in the same 5/6 nephrectomy model used a dietary approach of 1% of THCu + polyenylphosphatidylcholine (PPC) incorporated into pelleted chow [11]. The chow was consumed ad lib for nine weeks and yielded average steady-state plasma levels of 24.0 ± 3.3 μg/mL. The higher levels in the 2018 study may reflect continuous drug exposure from unrestricted dietary intake and the longer treatment duration. Our 2023 study in a rat diabetic nephropathy model employed once-daily oral gavage with PPC at 80 mg/kg for four weeks [49] with serum THCu measured at 2.7 ± 0.3 ng/mL (unpublished data). Collectively, these three studies demonstrate that oral THCu delivery can achieve measurable systemic levels and produce renoprotective effects.

Several limitations in this study warrant consideration. It is well known that for the same analysis task different bioinformatics tools could yield different results. The deconvolution software limma-voom used here is published and accepted in the field [64]. We acknowledge the limitations of using the mouse single-cell reference dataset for rat tissue deconvolution, which introduces potential cross-species bias; however, it is established that broad cell types are conserved between rat and mouse [65]. We emphasize that the resulting DEGs and pathway analysis are deemed exploratory. There was a discordance between transcriptomic remodeling (most significant with T500 treatment) and proteinuria reduction (most pronounced with T100 treatment)—though the difference in proteinuria between the three THCu treatment groups did not reach statistical significance. The absence of a monotonic dose–response relationship may indicate complex pharmacodynamic effects; conversely, transcriptomic remodeling may precede detectable functional recovery, and longer duration studies will be necessary to determine whether the molecular changes observed translate into measurable renal functional benefit. The study was conducted exclusively in female rats, which were selected based on their tendency to develop more pronounced proteinuria and renal fibrosis in the 5/6 nephrectomy model compared to males [31]. However, sex-specific hormonal factors may independently influence fibrosis pathways and transcriptional responses, and their contribution was not evaluated. The study did not examine THCu in healthy controls and lacked a vehicle control group. However, the liposomal carrier components Captex 300 (medium-chain triglycerides), Phosal 75 SA (phosphatidylcholine), and Labrasol ALF are established pharmaceutical excipients with established safety profiles in rodent studies [66,67,68]. Finally, we again emphasize that this study is exploratory, and confirmation of the metabolic and fibrosis alterations identified by the exploratory transcriptomic pathway analysis will require future protein-level studies in larger cohorts to clarify restoration of normal physiology vs. adaptive stress responses.

## 5. Conclusions

In conclusion, liposomal oral THCu therapy produced marked transcriptomic effects in a rat CKD model, including suppression of immune activation and kidney injury genes and improvement of renal metabolic and transport pathways. There was a reduction in proteinuria while kidney function markers were unchanged at the 4-week timepoint. Future longer-duration studies utilizing protein-level and functional assays are needed to validate the findings before moving into clinical translational trials.

## Figures and Tables

**Table 1:** Serum and urine chemistries, and tail blood pressure data in control (CTL) and chronic kidney disease (CKD) rat experimental groups. BP = tail blood pressure; BUN = blood urea nitrogen; CrCL = 24 h urine creatinine clearance; T100 = tetrahydrocurcumin, 100 mg/kg, twice per day; T300 = tetrahydrocurcumin, 300 mg/kg, twice per day; T500 = tetrahydrocurcumin, 500 mg/kg, twice per day. Data presented as mean ± standard error; * *p* < 0.05 compared with CTL; # *p* < 0.05 compared with untreated CKD.

Variable	CTL *n* = 6	CKD *n* = 9	CKD + T100 *n* = 8	CKD + T300 *n* = 9	CKD + T500 *n* = 9
Systolic BP	115.4 ± 6.4	134.1 ± 3.9	141.4 ± 4.5	130.7 ± 5.0	128.8 ± 9.1
Diastolic BP	79.6 ± 4.0	93.7 ± 3.1	98.4 ± 3.1	83.1 ± 3.7	80.6 ± 7.7
THCu serum level (ng/mL)	not detected	not detected	112 ± 27	1010 ± 292	5505 ± 3631
Creatinine (mg/dL)	0.27 ± 0.01	0.57 ± 0.04	0.62 ± 0.02 *	0.63 ± 0.03 *	0.60 ± 0.02 *
BUN (mg/dL)	17.3 ± 1.4	41.8 ± 2.0 *	43.5 ± 2.8 *	39.8 ± 2.3 *	35.6 ± 1.8 *
24 h urine protein excretion (mg/day)	1.2 ± 0.4	95.9 ± 25.8 *	11.8 ± 4.5 #	30.5 ± 12.1	39.1 ± 14.7
CrCL (mL/min × kg)	6.2 ± 0.4	2.9 ± 0.3 *	3.4 ± 0.3 *	2.8 ± 0.2 *	3.2 ± 0.1 *

**Table 2:** Number of differentially expressed genes (DEGs) across group comparisons and the global correlation value between replicate pairs, before and after accounting for cell-type proportions in the regression model.

Number of DEGs	CKD vs. CTL	T100 vs. CKD	T300 vs. CKD	T500 vs. CKD	Global R^2^
Without Cell Types	299	0	0	300	0.8676
With Cell Types	2108	15	27	203	0.7516

## Data Availability

The analyzed and raw data from the current study are available from the corresponding author upon request.

## Supplementary Materials

The following supporting information can be downloaded at: https://www.mdpi.com/article/10.3390/biomedicines14071457/s1, Figure S1: Exploratory MDS plots showed poor agreement between lane replicates of sample D6 from T300 group (left figure); MDS plot after rejecting sample D6 showed significant improvement in lane-wise correlation (right figure); Figure S2: Principal component regression was conducted on cell-type proportions and then correlated to the principal components (PCs) of the expression matrix. The majority cell types captured were proximal tubule and loop of Henle cells.

## Abbreviations

The following abbreviations are used in this manuscript:

Abcb11	ATP binding cassette subfamily B member 11
ANOVA	Analysis of variance
BP	Blood pressure
BUN	Blood urea nitrogen
Ccl5	C-C motif chemokine ligand 5
CD.IC	Intercalated cells of collecting duct
CD.PC	Principal cells of collecting duct
Cd53	CD53 molecule
CKD	Chronic kidney disease
CNT	Connecting tubule
Col1a1	Collagen type I alpha 1 chain
Col3a1	Collagen type III alpha 1 chain
Cr	Creatinine
CrCL	Creatinine clearance
CTL	Control
Cxcl13	C-X-C motif chemokine ligand 13
DAVID	Database for Annotation, Visualization, and Integrated Discovery
DCT	Distal collecting tubule
DEGs	Differentially expressed genes
Dnase1	Deoxyribonuclease 1
EC	Endothelial cells
FC	Fold-change
Gls2	Glutaminase 2
Gpx1	Glutathione peroxidase 1
GRTH	Genomics Research and Technology Hub
Gstm1	Glutathione S-transferase mu 1
Havcr1	Hepatitis A virus cellular receptor 1
Hmox1	Heme oxygenase 1
HPC	High-Performance Computing
HPLC-MS/MS	High-performance liquid chromatography-tandem mass spectrometry
Kim1	Kidney injury molecule-1
Lcp1	Lymphocyte cytosolic protein 1
LOH	Loop of Henle
Macro	Macrophages
MDS	Multidimensional scaling
Mes	Mesangial cells
MW	Molecular weight
PCs	Principal components
Pod	Podocytes
PPC	Polyenylphosphatidylcholine
PT	Proximal tubule
QC	Quality control
RGD	Rat Genome Database
S	Serum
SEM	Standard error of the mean
Slamf9	SLAM family member 9
T100	Tetrahydrocurcumin 100 mg/kg
T300	Tetrahydrocurcumin 300 mg/kg
T500	Tetrahydrocurcumin 500 mg/kg
THCu	Tetrahydrocurcumin
Timp1	Tissue inhibitor of metalloproteinases 1
U	Urine
UPLC	Ultra-performance liquid chromatography
V	Volume
W	Weight
