# Supplementary material for: Transcriptomic Exploration of Tetrahydrocurcumin Effects in Chronic Kidney Disease
Source: Biomedicines. 2026 Jun 26;14(7):1457. doi: 10.3390/biomedicines14071457 (PMC13405566; doi:10.3390/biomedicines14071457)
Supplement: Supplementary file 1 [file biomedicines-14-01457-s001.zip › Suppl Data THCu transcriptomics_v.4.27.2026.pdf]

Supplemental Data

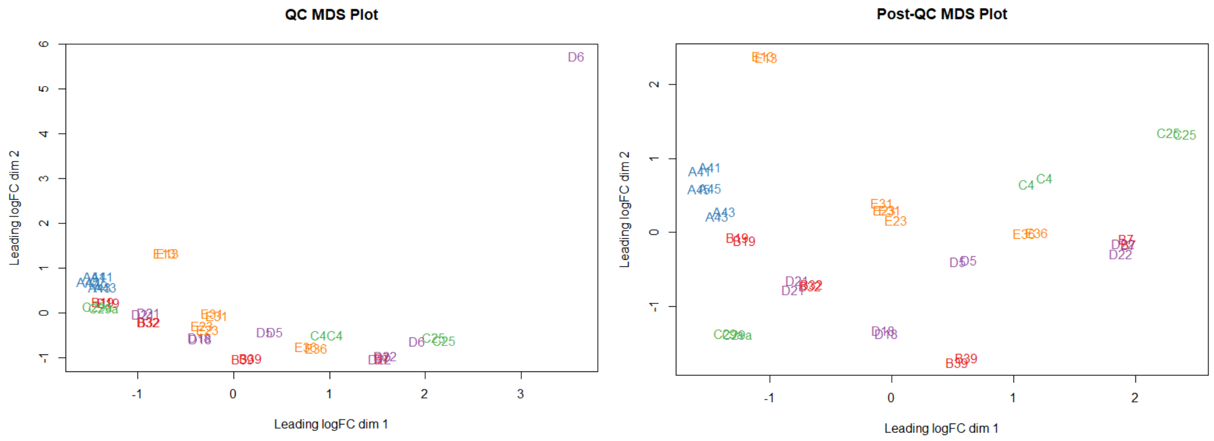

Figure S1. Exploratory MDS plots showed poor agreement between lane replicates of sample D6 from T300 group (left figure); MDS plot after rejecting sample D6 showed significant improvement in lane-wise correlation (right figure).

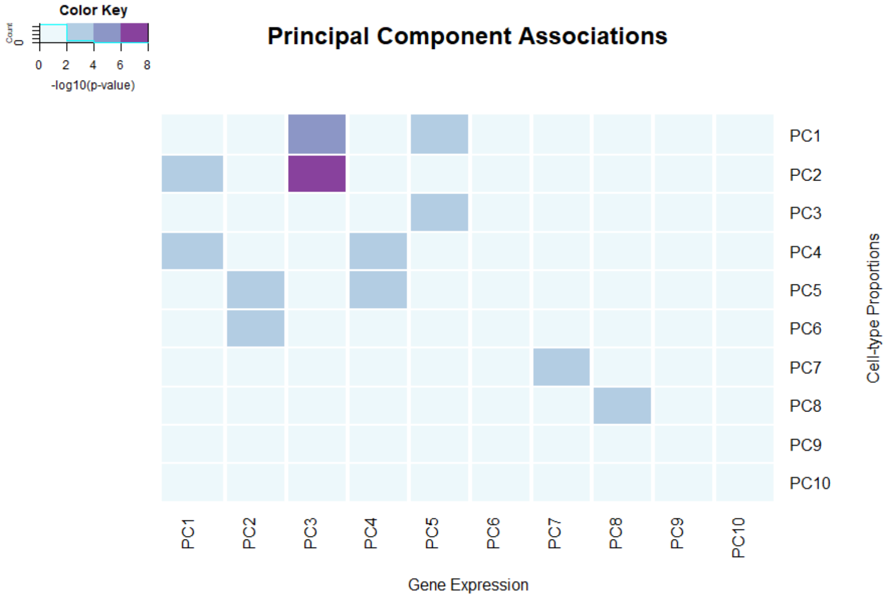

Figure S2. Principal component regression was conducted on cell-type proportions and then correlated to the principal components (PCs) of the expression matrix. The majority cell types captured were proximal tubule and loop of Henle cells.
